# Development of Injection-Molded Polylactide Pieces with High Toughness by the Addition of Lactic Acid Oligomer and Characterization of Their Shape Memory Behavior

**DOI:** 10.3390/polym11122099

**Published:** 2019-12-14

**Authors:** Diego Lascano, Giovanni Moraga, Juan Ivorra-Martinez, Sandra Rojas-Lema, Sergio Torres-Giner, Rafael Balart, Teodomiro Boronat, Luis Quiles-Carrillo

**Affiliations:** 1 Technological Institute of Materials (ITM), Universitat Politècnica de València (UPV), Plaza Ferrándiz y Carbonell 1, 03801 Alcoy, Spain; dielas@epsa.upv.es (D.L.); giovannimoraga92@gmail.com (G.M.); juaivma@epsa.upv.es (J.I.-M.); sanrole@epsa.upv.es (S.R.-L.); rbalart@mcm.upv.es (R.B.); tboronat@dimm.upv.es (T.B.); luiquic1@epsa.upv.es (L.Q.-C.); 2 Escuela Politécnica Nacional, 17-01-2759 Quito, Ecuador; 3 Novel Materials and Nanotechnology Group, Institute of Agrochemistry and Food Technology (IATA), Spanish National Research Council (CSIC), Calle Catedrático Agustín Escardino Benlloch 7, 46980 Paterna, Spain

**Keywords:** PLA, OLA, impact modifier, shape memory, packaging applications

## Abstract

This work reports the effect of the addition of an oligomer of lactic acid (OLA), in the 5–20 wt% range, on the processing and properties of polylactide (PLA) pieces prepared by injection molding. The obtained results suggested that the here-tested OLA mainly performs as an impact modifier for PLA, showing a percentage increase in the impact strength of approximately 171% for the injection-molded pieces containing 15 wt% OLA. A slight plasticization was observed by the decrease of the glass transition temperature (T_g_) of PLA of up to 12.5 °C. The OLA addition also promoted a reduction of the cold crystallization temperature (T_cc_) of more than 10 °C due to an increased motion of the biopolymer chains and the potential nucleating effect of the short oligomer chains. Moreover, the shape memory behavior of the PLA samples was characterized by flexural tests with different deformation angles, that is, 15°, 30°, 60°, and 90°. The obtained results confirmed the extraordinary effect of OLA on the shape memory recovery (R_r_) of PLA, which increased linearly as the OLA loading increased. In particular, the OLA-containing PLA samples were able to successfully recover over 95% of their original shape for low deformation angles, while they still reached nearly 70% of recovery for the highest angles. Therefore, the present OLA can be successfully used as a novel additive to improve the toughness and shape memory behavior of compostable packaging articles based on PLA in the new frame of the Circular Economy.

## 1. Introduction

Polylactide (PLA) is a linear thermoplastic biodegradable polyester that can be obtained from starch-rich materials by fermentation to give lactide, which is polymerized at the industrial scale by ring-opening polymerization (ROP) [1]. PLA is a sound candidate to substitute some plastic commodities such as polypropylene (PP) or polystyrene (PS) in packaging applications [2,3,4,5,6], electronics [7], automotive [8], agriculture [9], textile, consumer goods, 3D printing applications [10,11,12,13], biomedical devices [14,15], pharmaceutical carriers [16,17], etc. The main advantage of PLA over other biopolymers is its relatively low cost and overall balanced properties and processability [18,19], resulting in compostable articles [20]. In 2018, PLA production represented 10.3% of the worldwide production capacity of bioplastics, reaching nearly 220,000 tons/year and it is estimated a growth around 60% by 2023 [21].

Although PLA is a very versatile biopolymer, it results in extremely brittle materials with very low elongation at break and low toughness [22]. Many research works have been focused on overcoming or, at least, minimizing the intrinsic brittleness of PLA materials. One possible strategy is copolymerization with long aliphatic monomers, as suggested by Zhang et al. [23]. This is the case of the new type of polyester amide (PEA) copolymers developed by Zou et al. [24] combining poly(L-lactic acid) (PLLA) and poly(butylene succinate) (PBS) flexible segments. A similar approach was developed by Lan et al. [25] based on PLA-*co*-PBS copolymers. Nevertheless, the most widely used approach is blending with other biopolymers due to its cost effectiveness. For instance, Garcia-Campo et al. [26,27] reported interesting toughening effects on ternary blends composed of PLA, poly(3-hydroxybutyrate) (PHB), and different rubbery polymers such as PBS, poly(butylene succinate-*co*-adipate) (PBSA) or poly(ε-caprolactone) (PCL). Recently, Sathornluck et al. [28] developed binary blends of PLA with epoxidized natural rubber (ENR), which can positively contribute to improving toughness due to the effect of the rubber phase finely dispersed in a brittle PLA matrix. Su et al. [29] and Zhang et al. [30] have also reported different approaches to overcome the intrinsic brittleness of PLA by adding different contents of PBS using reactive compatibilizers. Fortelny et al. [31] recently reported, however, that it is possible to obtain “super-toughened” PLA formulations by blending PLA with poly(ε-caprolactone) (PCL) without any compatibilizer but using the appropriate PCL particle size. Addition of modified or unmodified particles is another way to overcome the intrinsic PLA brittleness. For instance, Wang et al. [32] improved the PLA toughness by means of silanized helical carbon nanotubes (CNTs). Similarly, Li et al. [33] reported the positive effect of cellulose nanofibers (CNFs) and a Surlyn^®^ ionomer to improve the interfacial interaction between CNFs and PLA, leading to a remarkable increase in toughness. The work carried out by Gonzalez-Ausejo et al. [34] reported a clear improvement in toughness by using sepiolite nanoclays as gas barrier and compatibilizer in PLA/poly(butylene adipate-*co*-terephthalate) (PBAT) blends.

Even though copolymerization and blending are some of the most used procedures to overcome the intrinsic brittleness of PLA, plasticization is another interesting approach. Plasticizers have been widely used in PLA formulations to reduce fragility by decreasing the glass transition temperature (T_g_). Plasticizers contribute to an increase in ductile properties, such as elongation at break, but mechanical resistant properties are also negatively decreased. Therefore, the use of plasticizers not always provides enhanced toughness since it depends on both ductile and resistant properties. For instance, Tsou et al. [35] improved toughness of PLA by using an adipate ester plasticizer. It has also been reported the positive effect of combining two plasticizers: one solid plasticizer, namely poly(ethylene glycol) (PEG), and a liquid plasticizer derived from soybean oil [36]. Indeed, many research works have been focused on using environmentally friendly plasticizers, which can contribute to improving toughness without compromising the overall biodegradability of PLA. In this regard, Kang et al. [37] described the usefulness of cardanol obtained from cashew nutshell liquid (CNSL) to obtain a 2.6-fold increase in toughness over neat PLA. Carbonell-Verdu et al. [38] developed a new series of dual plasticizer and compatibilizer additives derived from cottonseed oil subjected to epoxidation and/or maleinization as an environmentally friendly solution instead of using conventional epoxy-styrene acrylic oligomers. Other similar multi-functionalized vegetable oils have demonstrated good compatibilizing effects on PLA [39,40] and also on its blends with other biopolymers [41]. Ferri et al. [42] recently reported a remarkable increase in toughness of neat PLA by using maleinized linseed oil (MLO) as a bio-based plasticizer, which yielded a slight plasticization that overlapped with chain extension, branching, and some cross-linking. Tributyl citrate (TBC) has also been proposed as an effective plasticizer for PLA by Notta-Cuvier et al. [43], who reported the synergistic effect of TBC in PLA formulations containing halloysite nanotubes (HNTs).

Oligomers of lactic acid (OLAs) can also provide plasticization to PLA and PLA-based materials [44]. As reported by Burgos et al. [45], OLAs can contribute to a significant decrease in T_g_, though their role as impact modifiers require further research. Therefore, this study focuses on the effect of the addition of a new type of OLA on PLA pieces prepared by injection molding. In particular, the effect of varying the OLA content on the mechanical, thermal, thermomechanical properties and the morphology of PLA was reported. Finally, the shape memory behavior of the pieces was analyzed and related to the OLA dispersion within the PLA matrix.

## 2. Experimental

### 2.1. Materials

PLA was supplied by NatureWorks LLC (Minnetonka, MN, USA) as Ingeo^TM^ Biopolymer 6201D. This PLA grade contains 2 mol% _D_-lactic acid. It was supplied in pellet form and it had a density of 1.24 g cm^−3^ whereas its melt flow index (MFI) was 20 g/10 min, measured at 210 °C and 2.16 kg. OLA was kindly supplied by Condensia Química S.A. (Barcelona, Spain) as Glyplast OLA 2. It was provided in a liquid form with a viscosity of 90 mPa.s at 40 °C. According to the manufacturer, it has an ester content >99%, a density of 1.10 g cm^−3^, a maximum acid index of 2.5 mg KOH g^−1^, and a maximum water content of 0.1 wt%.

### 2.2. Manufacturing of OLA-Containing PLA Pieces

As PLA is very sensitive to moisture, the biopolyester pellets were dried at 60 °C for 24 h. The OLA content varied in the 0–20 wt% at weight steps of 5 wt%. The terminology used for the formulations was “PLA-OLA x%”, where x represents the weight fraction of OLA in PLA. All the compositions were compounded in a twin-screw co-rotating extruder Coperion ZS-B 18 (Stuttgart, Germany) equipped with a main hopper in which the PLA pellets were fed and a side feeder for liquids to feed OLA. The liquid feeder was heated at 50 °C during compounding to decrease the OLA viscosity and allow efficient mixing. The rotating speed was set to 150 rpm while the temperature profile was modified according to the formulation as shown in Table 1. These temperatures were selected due to the change in viscosity produced by the OLA addition in order to optimize the processing conditions for each formulation. After extrusion, the different strands were cooled in air and then pelletized using an air-knife unit.

The compounded pellets were stored in an air-circulating oven at 60 °C for 24 h to avoid moisture gain. Standard samples for characterization were thereafter obtained in an injection molding machine Meteor 270/75 from Mateu & Solé (Barcelona, Spain). The temperature profile of the four barrels was programmed as indicated in Table 2. Similar to the extrusion process, it was necessary to optimize the temperature profile for each formulation due to the drop in the melt viscosity after the OLA addition.

### 2.3. Mechanical Characterization

Mechanical properties of the OLA-containing PLA pieces were obtained in tensile conditions as indicated by ISO 527-1:1996. A universal testing machine ELIB 30 from S.A.E. Ibertest (Madrid, Spain) was used. At least six different samples were tested at room temperature using a load cell of 50 kN. The cross-head speed rate was set to 10 mm min^−1^. As recommended by the standard, the tensile modulus (E_t_), tensile strength at break (σ_b_), and elongation at break (%ε_b_) were determined and averaged. The toughness was estimated using the Charpy method following the guidelines of ISO 179-1:2010 with a 6-J pendulum from Metrotec (San Sebastián, Spain). Unnotched rectangular samples with dimensions 80 × 10 mm^2^ and a thickness of 4 mm were tested. The impact strength was obtained from testing five different samples and calculated as the absorbed-energy per unit area (kJ m^−2^). The Shore D hardness was determined in a 673-D durometer from J. Bot Instruments (Barcelona, Spain) according to ISO 868:2003. Ten different measurements were collected from randomly selected zones and various samples were tested to obtain the average values.

### 2.4. Microscopy

The morphology of the fracture surfaces of the OLA-containing PLA pieces was studied by field emission scanning electron microscopy (FESEM) after the impact tests. A ZEISS Ultra 55 FESEM microscope from Oxford Instruments (Abingdon, UK) operating at 2 kV was used to collect the FESEM images at 1000×. To avoid electrical charging during observation, samples were previously coated with an ultrathin gold-palladium alloy in an EMITECH SC7620 sputter-coater from Quorum Technologies Ltd. (East Sussex, UK) in an argon atmosphere.

### 2.5. Thermal Characterization

Thermal properties of the OLA-containing PLA pieces were obtained by differential scanning calorimetry (DSC) and thermogravimetric analysis (TGA). DSC characterization was performed on a Mettler-Toledo DSC calorimeter DSC821e (Schwerzenbach, Switzerland). To carry out the DSC runs, small specimens of each composition with an average weight of 5–7 mg were placed in standard aluminum crucibles with a total volume of 40 mL and sealed with a cap. Then, the samples were subjected to a three-step temperature program. A first heating cycle from 30 °C to 200 °C was followed by a cooling step down to −60 °C and, finally, a second heating cycle from −60 °C to 350 °C was applied. The heating and cooling rates were set to 10 °C min^−1^ whereas the selected atmosphere was nitrogen at a flow-rate of 30 mL min^−1^. The resultant DSC curves allowed obtaining T_g_, the cold crystallization peak temperature (T_cc_), and the melting peak temperature (T_m_). Besides, the cold crystallization (Δ*H_cc_*) and melting enthalpies (Δ*H_m_*) were obtained from the integration of the corresponding peaks. The maximum degree of crystallinity (%*χ*_cmax_) was calculated as indicated in Equation (1):

(1) $$\%\chi_{cmax}=\frac{\Delta H_{m}}{\Delta H_{m}^{0}}\cdot \frac{100}{w}$$

where *w* (g) stands for the weight fraction of PLA and Δ*H_m_^o^* (J g^−1^) represents the theoretical melting enthalpy of a fully crystalline PLA polymer, which is close to 93.7 J g^−1^ [46,47,48,49].

The effect of the OLA addition on the thermal stability of PLA was studied by TGA in a TGA/SDTA 851 thermobalance from Mettler-Toledo (Schwerzenbach, Switzerland). The temperature sweep was scheduled from 30 °C to 700 °C at a heating rate of 10 °C min^−1^ in air atmosphere. Samples with an average weight comprised between 5 and 7 mg were placed on standard alumina crucibles and sealed with the corresponding cap. The onset degradation temperature, which was assumed at a weight loss of 5 wt% (T_5%_), and the maximum degradation rate temperature (T_deg_) were obtained. All DSC and TGA tests were run in triplicate to obtain reliable results.

### 2.6. Viscosity Characterization

To study the influence of the OLA addition on the viscosity of PLA, cylindrical disks sizing 40 mm diameter and 5 mm thickness were manufactured by hot-press molding in a BUEHLER SimpliMet 1000 (Lake Bluff, IL, USA) at a temperature of 165 °C and a pressure of 7.5 ton. Parallel-plate oscillatory rheometry (OR) was conducted in an AR-G2 rheometer from TA Instruments (New Castle, DE, USA) to obtain the evolution of the complex viscosity (|η*|) as a function of the angular frequency. The selected isothermal temperature was 200 °C and the angular frequency varied in the 100–0.01 rad s^−1^ range. The maximum shear strain (γ) was set to 1% and the tests were carried out in air atmosphere in triplicate.

### 2.7. Thermomechanical Characterization

The effect of the OLA addition on the thermomechanical behavior of PLA was carried out by dynamic mechanical thermal analysis (DMTA) in a DMA 1 from Mettler-Toledo (Schwerzenbach, Switzerland) working in single cantilever flexural conditions. Rectangular samples with dimensions of 10 × 7 mm^2^ and a thickness of 4 mm were subjected to a dynamic heating program from −30 °C to 130 °C at a constant heating rate of 2 °C min^−1^. The maximum deflection in the free edge was set to 10 μm and the selected frequency was 1 Hz. The storage modulus (E*’*) and the dynamic damping factor (*tan δ*) were collected as a function of increasing temperature.

The dimensional stability of the OLA-containing PLA pieces was studied by thermomechanical analysis (TMA) in a Q400 thermomechanical analyzer from TA Instruments (New Castle, DE, USA). The applied force was set to 0.02 N and the temperature program was scheduled from −30 to 120 °C in air atmosphere (50 mL min^−1^) at a constant heating rate of 2 °C min^−1^. The coefficient of linear thermal expansion (CLTE) of the PLA pieces, both below and above T_g_, was determined from the change in dimensions versus temperature.

### 2.8. Characterization of the Shape Memory Behavior

The flexural method was used to evaluate the extension of the shape memory behavior. To this end, samples were compression-molded into sheets in the hot-press molding with a thickness of 0.4–0.5 mm. Two different parameters were then calculated to analyze the shape memory behavior in flexural conditions, namely the shape memory recovery (*R_r_*) and stability ratio (*R_f_*) [50,51]. The procedure is described as follows. In the first stage, the sheet specimens were deformed at a particular angle *θ_f_*. For this, the samples were heated at 65 °C and forced to adapt between two aluminum sheets with different angles (15°, 30°, 60°, and 90°) to form a sandwich structure: aluminum/PLA sheet/aluminum. The sandwich was then clamped to allow a permanent deformation to the desired angle. Thereafter, the sandwich was cooled down to 14 °C to achieve its permanent shape after a slight temporary recovery of *θ_t_*. Finally, the specimen was heated above its T_g_ in an air circulating oven at 65 °C for 3 min. After this, the final angle, that is, *θ_f_*, was measured. At least three different sheets were tested for each composition to obtain reliable values. The same procedure has been used to characterize the shape recovery behavior in several polymer systems [52,53]. The values of *R_f_* and *R_r_* were calculated using the following equations:

(2) $$\%R_{f}=\frac{\left(\pi -\theta_{t}\right)}{\left(\pi -\theta_{f}\right)}.$$

(3) $$\%R_{r}=\frac{\left(\pi -\theta_{f}\right)/\left(\pi -\theta_{r}\right)}{\left(\pi -\theta_{f}\right)}.$$

## 3. Results and Discussion

### 3.1. Effect of OLA on the Mechanical Properties of PLA

Table 3 gathers the main results obtained after the mechanical characterization of the PLA pieces with different OLA contents. As expected, the progressive addition of OLA led to lowering σ_b_ values from 64.6 MPa, for the neat PLA, down to 37.4 MPa, for the PLA pieces containing 20 wt% OLA. This decreasing tendency was almost linear as it can be seen in the table for the other compositions. This tendency on mechanical strength is the typical a plasticizer produces on the base polymer. Some other OLA additives have demonstrated a similar effect on mechanical properties by increasing remarkably ductility, mainly in the film form, as reported by Burgos et al. [45]. In the latter work, it was reported an increase in ε_b_ from 4% to 315% with an OLA content of 25 wt%, but it is worthy to note the OLA previously used was designed for plasticization of PLA films. Such dramatic increase in the ε_b_ was not observed when using OLA in this work since the primary use of this type of OLA was to improve impact strength, as indicated by the supplier and it will be discussed further.

Regarding mechanical ductility, the neat PLA piece showed a very low value of 7.9% and the addition of OLA did not promote an increase in ε_b_, but a slight decrease down to values of 5%. It is not usual that a plasticizer promotes a decrease in ductility since the typical effect of a plasticizer is a decrease in the tensile resistant properties (σ_b_ and E_t_) and an increase in ductile properties (ε_b_). Nevertheless, it has been reported that some plasticizers promote a clear plasticization that is detectable by a decrease in T_g_ while no improvement in ductility occurs. This atypical behavior was reported by Ambrosio-Martín et al. [54] in PLA films blended with different synthesized OLAs. A ε_b_ value of 5.25% was reported for neat PLA, while the addition of 25 wt% of a purified OLA yielded a ε_b_ of 2.52%. It was also reported a slight increase in E_t_ and an apparent decrease in σ_b_, in a similar way as obtained in this work. It was concluded that, although there is clear evidence of the mechanical plasticization of OLA-containing PLA films, they were not more deformable, which is also in agreement with the work performed by Courgneau et al. [55]. Concerning E_t_, the neat PLA piece was characterized by a value of nearly 2.2 GPa and the values remained in the 2.2–2.4 GPa range after the addition of OLA. Thus, the main effect of this type of OLA on the tensile mechanical properties was a remarkable decrease in σ_b_, which representative for some plasticization, but also a slight decrease in ε_b_. Interestingly, as it can also be seen in Table 3, the addition of OLA successfully increased the impact strength of PLA. Neat PLA showed an impact strength of 25.7 kJ m^−2^, which indicates a brittle behavior, and the only addition of 5 wt% OLA provided a slight increase in the impact strength to 30.4 kJ m^−2^. Nevertheless, the most remarkable changes were obtained for OLA additions of 10 wt% and 15 wt%, showing impact strength values of 54.2 kJ m^−2^ and 69.7 kJ m^−2^, respectively. Therefore, the PLA piece with 15 wt% OLA presented the maximum impact strength with a percentage increase of approximately 171% with regard to the neat PLA. It is also worthy to mention that the PLA piece containing 20 wt% OLA showed a decrease in impact strength in comparison with the other OLA-containing PLA pieces, thus suggesting certain OLA saturation in the PLA matrix. In this regard, Fortunati et al. [56] reported the use of isosorbide diester (ISE) as plasticizer for PLA. It was observed plasticizer saturation at 20 wt% ISE and this was attributed to a limitation of T_g_ decrement. In addition, a noticeable decrease in ε_b_ was observed once the plasticizer saturation was achieved. Furthermore, Ferri et al. [57] reported the plasticization of PLA by fatty acid esters, observing a remarkable decrease in impact strength above 5 parts per one hundred parts (phr) of PLA. Accordingly, a remarkable decrease in T_g_ was attained for contents of up to 5 phr whereas, above this, the T_g_ values did not change in a noticeable way, corroborating the relationship between the impact and thermal properties. Regarding hardness, one can observe that the Shore D values remained nearly constant after the OLA addition, showing values in the 78–82 range. Therefore, the most important feature this OLA can potentially provide to the mechanical properties of PLA is a remarkable improvement in impact strength while the elasticity can be slightly improved and ductility reduced. This particular mechanical behavior could be ascribed to an increase in the sample crystallinity and also to the presence of soft domains of OLA dispersed within the PLA matrix, simultaneously improving impact strength and reducing flexibility.

**Figure 1:**
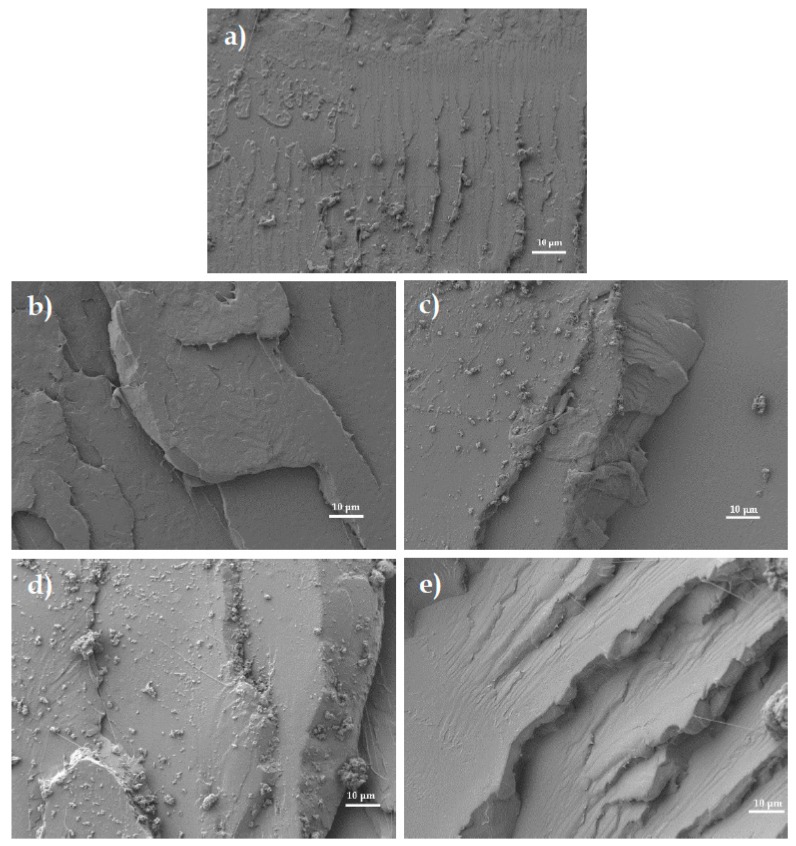
Field emission scanning electron microscopy (FESEM) images of the fracture surfaces of the of the polylactide (PLA) pieces with different weight contents of oligomer of lactic acid (OLA): (**a**) 0 wt%; (**b**) 5 wt%; (**c**) 10 wt%; (**d**) 15 wt%; and (**e**) 20 wt%. Images were taken at 1000× and scale markers are of 10 µm.

As previously indicated, one of the most widely used strategies to improve toughness in PLA-based formulations is blending with rubber-like polymers such as PCL [58], PBS [29,59], or PBAT [60,61]. In these immiscible blends, the energy absorption is related to presence of finely dispersed rubber-like small polymer droplets embedded in the brittle PLA matrix. In some cases, a synergistic effect can be found when different reactive or non-reactive compatibilizers are used. In this work, OLA has the same chemical structure than PLA, thus leading to miscibility without the need of compatibilizers. In this regard, Burgos et al. [45] have reported the similarity between the solubility parameters of both PLA and OLA, which plays an essential role in miscibility. According to this, Figure 1 gathers the FESEM images corresponding to the fracture surfaces from the impact tests of the PLA pieces with the different OLA loadings. Figure 1a shows the fracture surface of the neat PLA piece. As one can observe in this micrograph, the surface was smooth with multiple microcracks presence, which is an indication of a brittle behavior. As opposite, Figure 1b shows that the fracture surface morphology of the PLA piece with 5 wt% OLA changed noticeably. Phase separation could not be detected due to the high chemical affinity between PLA and OLA and the microcracks were not observed but, in contrast, macrocracks were produced. Therefore, the presence of OLA seems to inhibit microcrack formation and growth and, therefore, the cracks could grow to a greater extent thus leading to a rougher surface that is responsible for higher energy absorption during impact. Figure 1c,d show the FESEM images corresponding to the fracture surfaces of the PLA pieces with 10 wt% and 15 wt% OLA, respectively. In these images, the above-mentioned effect was more intense then showing rougher surfaces that are related to enhanced energy absorption. Finally, Figure 1e shows that the PLA piece containing 20 wt% OLA presented a similar fracture surface than the other pieces and, despite there was a clear loss of toughness, its morphology did not allow identifying phase separation.

### 3.2. Effect of OLA on the Thermal and Rheological Properties of PLA

**Figure 2:**
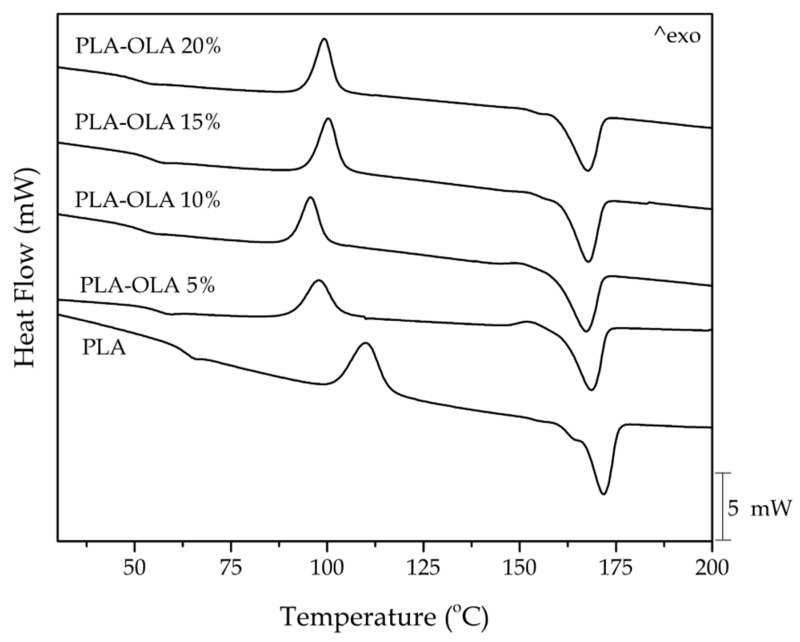
Differential scanning calorimetry (DSC) thermograms corresponding to the polylactide (PLA) pieces with different weight contents of oligomer of lactic acid (OLA).

Figure 2 shows a comparative graph with the characteristic DSC thermograms during first heating corresponding to the neat PLA piece and the PLA pieces with different OLA loadings. Table 4 summarizes the main thermal values obtained from the thermograms. One can observe that T_g_ of neat PLA was located close to 63 °C. Then, the PLA sample cold crystallized indicating that the biopolyester chains could not crystallize in the injection mold. The process of cold crystallization was characterized by a peak at 109.8 °C. Finally, the melting process was defined by a peak temperature of 170.9 °C in which the whole crystalline fraction melted. The effect of the OLA addition on the thermal properties was remarkable. Concerning T_g_, a clear decreasing tendency can be observed. In particular, T_g_ was reduced after the OLA addition down to 50.8 °C, thus indicating plasticization. Ambrosio-Martín et al. [54] reported a decrease in T_g_ with addition of OLA to PLA films from 60 °C to 27.7 °C, concluding that this fact is directly related to the mechanical properties of OLA and it depends on the synthesis procedure [62]. In the present work, the T_g_ value of PLA decreased after the addition of OLA but this reduction was much lower than other reported to other plasticizers. For instance, Ljungberg et al. [63] reported a T_g_ decrease of 30 °C with 15 wt% addition of different plasticizers such as triacetin, tributyl citrate (TBC), triethyl citrate (TEC), acetyl tributyl citrate (ATBC), and acetyl triethyl citrate (ATEC).

Furthermore, the added OLA induced an internal lubricating effect that shifted the cold crystallization of PLA to lower temperatures due to an increase of chain mobility. Then T_cc_ lowered to values in the range of 96–101 °C with the different OLA loadings. Other authors suggested a specific nucleating effect provided by the short length OLA molecules, which are more readily to pack the PLA macromolecular structure thus favoring the cold crystallization process [64]. In addition, a small and broad exothermic peak was seen in the PLA sample processed with OLA, particularly noticeable at the lowest OLA contents. This exothermic peak is related to a pre-melt crystallization just before melting. In this regard, one can consider that the presence of OLA promoted the formation of different crystallites [63]. As reported by Maróti et al. [65] and also Maiza et al. [66], this peak has been observed in neat PLA depending on the heating rate and the applied thermal cycle. One can also observe a slight decrease in the T_m_ value with the increasing OLA content. Similar findings have been reported by Burgos et al. [62] in PLA films with different OLAs.

In addition to the characteristic values of T_g_, T_cc_, and, T_m_, the enthalpies corresponding to the cold crystallization and melting processes, that is, Δ*H_cc_* and Δ*H_m_*, respectively, were collected. The maximum degree of crystallinity, that is, *χ_cmax_*, which does not consider the amount of crystals formed during cold crystallization, was 35.6% for the neat PLA. Then, *χ_cmax_* increased up to values of around 50% for the compositions containing 5–15 wt% OLA, while slightly lower values of crystallinity were obtained for the composition containing 20 wt% OLA, that is, 45.8%. This increase in crystallinity can be related to the plasticizing effect of OLA, as earlier reported by Burgos et al. [62] in PLA formulations with 15 wt% of different OLAs. This latter study also reported a decrease in the T_m_ value of approximately 5 °C.

**Figure 3:**
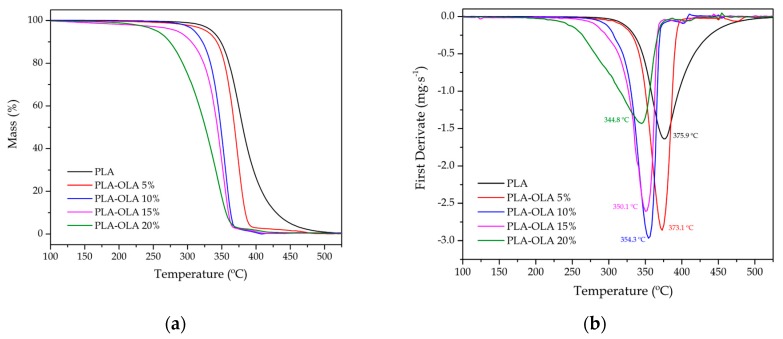
(**a**) Thermogravimetric analysis (TGA) and (**b**) first derivate thermogravimetric (DTG) curves corresponding to the polylactide (PLA) pieces with different weight contents of oligomer of lactic acid (OLA).

Regarding thermogravimetric characterization, Figure 3 shows the mass versus temperature (Figure 3a) and the first derivative (DTG) versus temperature (Figure 3b) curves for all the PLA pieces. The main results of the thermal decomposition of PLA-OLA blends are summarized in Table 5. One can observe that the neat PLA was much more thermally stable than the toughened PLA formulations with the different OLA loadings. As the OLA content increased, the characteristic TGA curves in Figure 3a shifted to lower temperatures, thus indicating a decrease in thermal stability. DTG curves were very useful to determine the maximum degradation rate temperature (T_deg_), which was seen as peaks in Figure 3b. It can be seen in the graph that there was a clear decreasing tendency of T_deg_ with increasing OLA content. Furthermore, the residual mass for all PLA formulations with OLA was almost the same, being below 1 wt%.

Therefore, OLA induced a remarkable reduction in the thermal stability of PLA, as similarly described by Ambrosio-Martín et al. [54]. In this regard, Burgos et al. [62] also reported individual TGA characterization of different OLAs with their corresponding thermal degradation parameters. A variation in the onset degradation temperature was observed from 179 °C to 214 °C. The lower thermal stability was related to the lower T_g_ values. Moreover, it was reported a maximum degradation rate temperature ranging from 259 °C to 291 °C. These characteristic degradation temperatures are remarkably lower than those of the neat PLA herein studied. As the OLA content in PLA pieces increased, both the T_5%_ and the T_max_ values showed a clear decreasing tendency. This decrease was more pronounced in the case of T_5%_, which varied from 336.1 °C, for the neat PLA piece, to 254.8 °C, for the PLA piece containing 20 wt% OLA.

**Figure 4:**
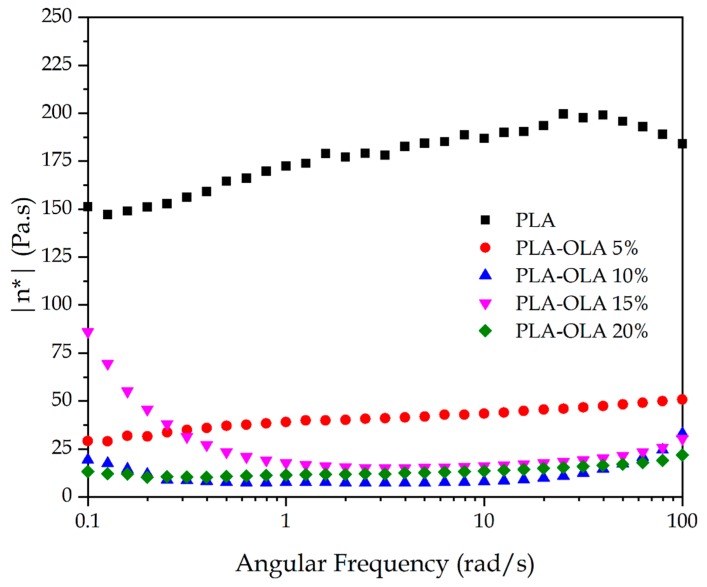
A comparative plot of the complex viscosity (|η*|) of the polylactide (PLA) sheets with different weight contents of oligomer of lactic acid (OLA) at a constant temperature of 200 °C as a function of increasing angular frequency.

As indicated previously, OLA provided a lubricating effect, which could potentially lead to a subsequent decrease in the viscosity. Figure 4 shows the effect of the different OLA loadings on the |η*| values of the PLA sheets as a function of the angular frequency. As it can be seen, the effect of the angular frequency on the complex viscosity was not highly pronounced but it was possible to detect a decreasing tendency of |η*| with increasing the OLA content. This confirms that OLA lowers the viscosity of the PLA melt during processing and it can therefore potentially act as a processing aid. In fact, as indicated previously, it was necessary to adjust the thermal profile for optimum processing when the OLA content was modified.

### 3.3. Effect of OLA on the Thermomechanical Properties of PLA

**Figure 5:**
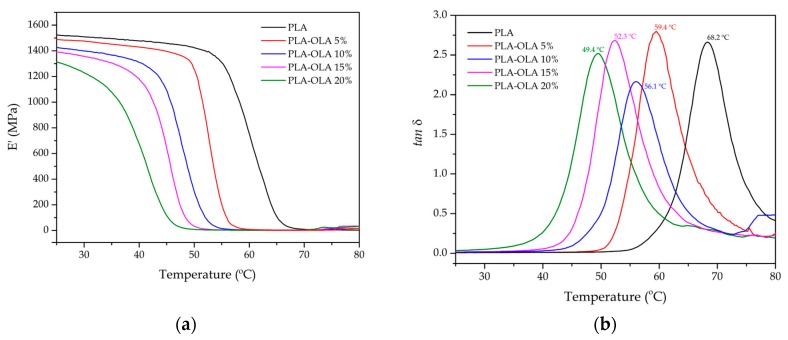
Evolution as a function of temperature of the (**a**) storage modulus (E’) and (**b**) dynamic damping factor (*tan δ*) of the polylactide (PLA) pieces with different weight contents of oligomer of lactic acid (OLA).

The results described above indicated a definite improvement in the PLA toughness by using OLA as an impact modifier. Mechanical characterization showed a decrease in mechanical strength while ductility was also slightly reduced. DMTA allows characterization of mechanical properties in dynamic conditions (sinusoidal applied stress) as a function of a heating cycle. Figure 5 shows the DMTA curves for the neat PLA piece and the PLA pieces containing different loadings of OLA. The results of the thermomechanical properties obtained by DMTA are summarized in Table 6. The variation of the E’ values of the neat PLA (Figure 5a) showed a dramatic drop between 50 °C and 70 °C, which is representative of the α-relaxation process of the PLA chains as the glass transition region was surpassed. In particular, a three-fold decrease in E’ was observed. As the OLA loading increased, the E’ curves shifted to lower temperatures thus indicating a decrease in T_g_, as previously observed by DSC analysis. In particular, the E’ values decreased from 1500 MPa, for the neat PLA piece, to 1197 MPa, for the PLA piece blended with 20 wt% OLA at 30 °C. As reported by other authors, both nucleating agents and plasticizers play a crucial role in the DMTA behavior of PLA [67,68]. Although the characteristic E’ curves showed a decrease in T_g_, more accurate values can be obtained by determining the peak maximum of *tan δ*, as observed in Figure 5b. Neat PLA showed a T_g_ of 68.2 °C and the T_g_ values decreased progressively as the OLA loading increased, reaching a minimum value of 49.4 °C for the PLA piece containing 20 wt% OLA. These results are in total agreement with the above-described results obtained during the DSC characterization.

Another attractive thermomechanical property is the effect of temperature on the dimensional stability of the PLA-based materials with different OLA loadings. TMA is a very useful technique to determine the CLTE values, which is a crucial property related to dimensional stability in terms of temperature exposition. Table 6 gathers these coefficients for the neat PLA piece and the PLA pieces containing different amount of OLA. As one can observe in the table, two different CLTE values were determined, one corresponding to the slope below T_g_ and another one corresponding to that above T_g_. Plasticization can be observed by seeing the CLTE both below and above T_g_. A slight increasing tendency was obtained, thus, indicating more ductility. The CLTE below T_g_ changed from 79.9 μm m^−1^ K^−1^ to 91.6 μm m^−1^ K^−1^. The maximum change was therefore 11.7 μm m^−1^ K^−1^, which is a very narrow range, typical of values below T_g_ then indicating excellent dimensional stability. Above T_g_, the maximum change was 29.1 μm m^−1^ K^−1^, which is in accordance with the typical plastic thermomechanical behavior above T_g_.

### 3.4. Effect of OLA on the Shape Memory Behavior of PLA

**Figure 6:**
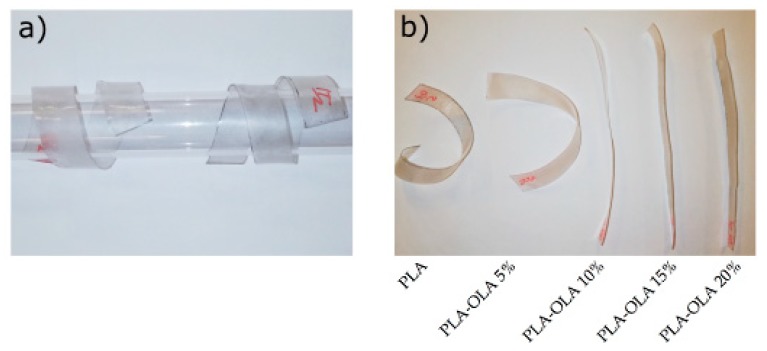
Photographs of the qualitative study of the shape memory recovery capacity of the polylactide (PLA) sheets with different weight contents of oligomer of lactic acid (OLA): (**a**) initial deformation of the sheets by introducing them into a glass tube and (**b**) recovered shape of the sheets after heating at 70 °C.

Initially, the shape memory behavior was studied qualitatively by introducing the sheet specimens into a glass tube at room temperature, as observed in Figure 6a, and remained inside for 5 min to retain the shape. Then, the crimped PLA sheets were immersed in a water bath at 70 °C, above the biopolyester’s T_g_, and allowed to recover their shape. As can it be seen in Figure 5b, flat sheet shapes were obtained for the PLA materials containing >10 wt% OLA loadings in a short period of 4–10 s, therefore giving support to the significant effect of OLA on the shape memory behavior of PLA. Similar results, under the same conditions, were reported in the development of poly(l-lactide-*co*-ε-caprolactone) (PLACL), which showed recovery times of approximately 20 s [69]. Another study was focused on PLA/thermoplastic polyurethane (TPU) blends in which the recovery times at 70 °C were very similar to those obtained in this work, that is, 7–12 s [50].

**Figure 7:**
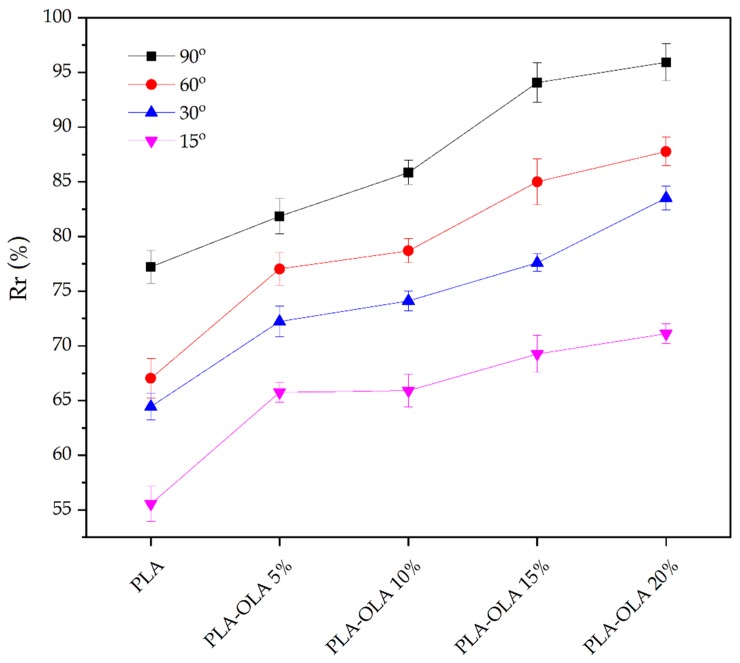
Evolution of the percentage of shape memory recovery (%*R_r_*) of polylactide (PLA) sheets with different weight contents of oligomer of lactic acid (OLA) at different initial deformation angles: 15°, 30°, 60°, and 90°.

In addition to the qualitative characterization shown above, a quantitative study was carried out to evaluate the influence of the OLA impact modifier on the shape memory behavior of PLA. Figure 7 shows the evolution of *R_r_* for different angles as the OLA content was increased. As it can be seen in the plot, the neat PLA sheet showed limited shape recovery properties and, obviously, it highly depended on the deformation angle. This shape memory recovery was close to 77% for a deformation angle of 90° and it was remarkably lower with more aggressive deformations. For instance, PLA could only recover 55.5% when the initial deformation angle was 15°. As the OLA loading increased, the ability of PLA to recover its initial flat shape (angle of 180°) increased considerably. It is worthy to note the effect of the addition of 20 wt% OLA, which yielded an almost constant increase in *R_r_* of approximately 20% for all the tested angles. Therefore, for this OLA loading, the shape recovery ability of PLA remarkably improved thus leading to an exciting shape memory behavior. As reported by Leonés et al. [70], a good shape memory behavior can be attained in electrospun PLA-based fibers containing different OLA loadings in the 10–30 wt% range.

The R_f_ value is representative for the dimensional stability below T_g_, after an initial deformation. As it can be seen in Table 7, R_f_ was very high for all the systems, thus indicating excellent dimensional stability after the first deformation. In general, as the deformation angle was lower, for instance 90º, the stability ratio was higher, showing recovery values over 98%. Alternatively, if the initial deformation angle was very aggressive, for instance 15°, a slight decrease in the stability ratio can be observed down to values of nearly 85%. Anyway, these stability ratios can be considered high for all compositions and angles. In this regard, Jing et al. [50] reported lower stability ratios in PLA/TPU blends, which showed less dimensional stability than the materials developed herein. Therefore, one can conclude indicating that this type of OLA is also an exceptional additive for shape memory recovery as shown by the high %R_r_ and %R_f_ values obtained.

## 4. Conclusions

The positive effect of OLA to improve PLA toughness was evaluated in this study. The addition of 15 wt% OLA provided an increase in the impact strength from 25.7 kJ m^−2^ to almost 70 kJ m^−2^, thus showing an extraordinary effect on toughness. Furthermore, the OLA impact modifier also provided a mechanical plasticization as observed by a decrease in the tensile strength though a slight reduction in ductility was also noticed. This plasticizing effect was observable by DSC in which the characteristic T_g_ of the neat PLA was reduced from 63.3 °C to 50.8 °C for the PLA piece with 20 wt% OLA. Moreover, since OLA was less thermally stable than PLA, a decrease in the onset degradation temperature, reported as T_5%_, was observed as the OLA loading increased. Nevertheless, the T_5%_ value corresponding to the PLA piece with the highest OLA loading was still high, that is, 254.8 °C, which successfully allows processing these blends without thermal degradation. Another exciting feature that this type of OLA can provide to PLA is the improvement of its shape memory behavior. In particular, the shape memory recovery parameter, that is, R_r_, was very high compared to other PLA-based blends or plasticized PLA systems, thus showing the extraordinary effect of this OLA on the shape memory recovery. As a general conclusion, the here-studied OLA additive represents an interesting technical and environmentally friendly solution to improve the intrinsic brittleness of PLA and it also contributes to somewhat plasticization that allows enhanced shape memory recovery properties. The resultant toughened PLA materials can be of high interest for the development of compostable packaging articles, such as food trays and films, or disposable articles, such as cutlery and straws.

## Figures and Tables

**Table 1:** Temperature profile of the seven barrels in the twin-screw extruder during the compounding of the polylactide (PLA)/oligomer of lactic acid (OLA) formulations.

PLA (wt%)	OLA (wt%)	T (°C) Zone 1	T (°C) Zone 2	T (°C) Zone 3	T (°C) Zone 4	T (°C) Zone 5	T (°C) Zone 6	T (°C) Zone 7
100	0	145	150	160	170	175	180	180
95	5	145	150	160	170	175	180	180
90	10	145	150	160	170	170	175	175
85	15	145	150	155	155	160	160	170
80	20	145	150	155	155	160	160	160

**Table 2:** Temperature profile of the four barrels and maximum pressure in the injection-molding machine during the manufacturing of polylactide (PLA)/oligomer of lactic acid (OLA) pieces.

PLA (wt%)	OLA (wt%)	T (°C) Zone 1	T (°C) Zone 2	T (°C) Zone 3	T (°C) Zone 4	Maximum Pressure (Bar)
100	0	175	180	185	190	93
95	5	175	180	185	190	90
90	10	160	165	170	175	118
85	15	150	155	165	170	120
80	20	145	150	155	165	125

**Table 3:** Mechanical properties of the polylactide (PLA) pieces with different weight contents of oligomer of lactic acid (OLA) in terms of: tensile modulus (E_t_), strength at break (σ_b_), elongation at break (ε_b_), impact strength, and Shore D hardness.

Piece	Tensile Test			Impact Strength (kJ m^−2^)	Shore D Hardness
	E_t_ (MPa)	σ_b_ (MPa)	ε_b_ (%)		
PLA	2251 ± 45	64.6 ± 1.1	7.9 ± 0.1	25.7 ± 2.7	78.8 ± 0.9
PLA-OLA 5%	2272 ± 94	52.0 ± 2.1	6.8 ± 0.3	30.4 ± 3.6	81.2 ± 1.4
PLA-OLA 10%	2300 ± 82	42.1 ± 1.7	5.6 ±0.2	54.2 ± 4.8	80.9 ± 2.3
PLA-OLA 15%	2400 ± 58	41.4 ± 1.0	5.3 ± 0.3	69.7 ± 5.2	80.9 ± 0.9
PLA-OLA 20%	2101 ± 91	37.4 ± 2.2	5.0 ± 0.2	38.4 ± 5.7	80.5 ± 0.4

**Table 4:** Thermal properties of the polylactide (PLA) pieces with different weight contents of oligomer of lactic acid (OLA) in terms of: glass transition temperature (T_g_), cold crystallization temperature (T_CC_), cold crystallization enthalpy (Δ*H_CC_*), melting temperature (T_m_), melting enthalpy (Δ*H_m_*), and degree of crystallinity (*χ_cmax_*).

Piece	T_g_ (°C)	T_cc_ (°C)	Δ*H_cc_* (J g^−1^)	T_m_ (°C)	Δ*H_m_* (J g^−1^)	*χ_cmax_* (%)
PLA	63.3 ± 1.5	109.8 ± 3.8	28.6 ± 0.8	170.9 ± 1.7	33.4 ± 1.6	35.6 ± 1.7
PLA-OLA 5%	55.9 ± 2.4	97.8 ± 2.1	22.5 ± 5.1	168.2 ± 2.4	46.2 ± 4.5	51.9 ± 4.7
PLA-OLA 10%	53.9 ± 1.9	100.1 ± 2.5	21.9 ± 1.7	167.1 ± 3.0	46.3 ± 4.8	54.9 ± 5.3
PLA-OLA 15%	51.3 ± 0.4	96.7 ± 2.1	26.3 ± 3.8	166.5 ± 2.1	42.4 ± 2.1	53.2 ± 2.5
PLA-OLA 20%	50.8 ± 3.2	99.1 ± 2.4	25.0 ± 2.5	166.9 ± 1.8	34.3 ± 3.2	45.8 ± 4.0

**Table 5:** Main thermal parameters of the polylactide (PLA) pieces with different weight contents of oligomer of lactic acid (OLA) in terms of: onset temperature of degradation (T_5%_), degradation temperature (T_deg_), and residual mass at 700 °C.

Piece	T_5%_ (°C)	T_deg_ (°C)	Residual Mass (%)
PLA	336.1 ± 2.7	375.9 ± 1.9	0.11 ± 0.02
PLA-OLA 5%	327.2 ± 1.2	373.1 ± 2.3	0.57 ± 0.08
PLA-OLA 10%	310.1 ± 3.2	354.3 ± 3.1	0.65 ± 0.12
PLA-OLA 15%	296.5 ± 3.8	350.1 ± 1.8	0.39 ± 0.08
PLA-OLA 20%	254.8 ± 2.8	344.8 ± 2.6	0.53 ± 0.07

**Table 6:** Main thermomechanical parameters of the polylactide (PLA) pieces with different weight contents of oligomer of lactic acid (OLA) in terms of: storage modulus (E’) measured at 30 °C and 70 °C, glass transition temperature (T_g_), and coefficient of linear thermal expansion (CLTE) below and above T_g_.

Piece	DMTA			TMA	
	E’ at 30 °C (MPa)	E’ at 70 °C (MPa)	T_g_ * (°C)	CLTE below T_g_ (μm m^−1^ K^−1^)	CLTE above T_g_ (μm m^−1^ K^−1^)
PLA	1500 ± 55	4.9 ± 0.3	68.2 ± 1.2	79.9 ± 3.5	155.4 ± 6.2
PLA-OLA 5%	1467 ± 49	2.4 ± 0.2	59.4 ± 1.4	87.1 ± 4.0	166.4 ± 7.9
PLA-OLA 10%	1389 ± 52	4.6 ± 0.4	56.1 ± 0.7	88.9 ± 3.8	173.8 ± 6.2
PLA-OLA 15%	1343 ± 38	5.2 ± 0.6	52.3 ± 0.9	90.1 ± 1.9	179.9 ± 7.1
PLA-OLA 20%	1197 ± 47	9.9 ± 1.1	49.4 ± 0.8	91.6 ± 3.9	184.5 ± 6.8

* T_g_ was obtained as the peak maximum of the dynamic damping factor (*tan δ*).

**Table 7:** Variation of the percentage of stability ratio (%R_f_) after different initial deformation angles (θ_f_) for the polylactide (PLA) sheets with different weight contents of oligomer of lactic acid (OLA).

Sheet	R_f_ (%)			
	θ_f_ = 90°	θ_f_ = 60°	θ_f_ = 30°	θ_f_ = 15°
PLA	99.6 ± 1.3	98.9 ± 2.1	99.6 ± 1.3	88.2 ± 1.1
PLA-OLA 5%	98.7 ± 1.2	98.1 ± 1.3	88.2 ± 1.3	87.2 ± 2.5
PLA-OLA 10%	98.4 ± 1.5	94.2 ± 2.4	96.8 ± 1.5	86.7 ± 1.3
PLA-OLA 15%	99.3 ± 1.4	98.1 ± 1.8	97.8 ± 2.1	85.2 ± 3.6
PLA-OLA 20%	91.4 ± 2.3	96.5 ± 2.4	98.4 ± 1.4	84.9 ± 1.6
